# Short-term patient-reported outcomes following total hip replacement: Is the success picture overrated?

**DOI:** 10.1016/j.ocarto.2021.100192

**Published:** 2021-06-15

**Authors:** Marek Kamil Gojło, Robert Lundqvist, Przemysław T. Paradowski

**Affiliations:** a Department of Orthopaedics and Traumatology, Ministry of Interior and Administration Hospital, Warmia and Mazury Oncology Center, Wojska Polskiego 37, PL-10-228, Olsztyn, Poland; b Research and Innovation Unit, Norrbotten County Council, SE-971 89, Luleå, Sweden; c Department of Surgical and Perioperative Sciences, Division of Orthopedics, Sunderby Research Unit, Umeå University, Sunderby Central Hospital of Norrbotten, SE-971 80, Luleå, Sweden; d Faculty of Health Sciences, Ludwik Rydygier Collegium Medicum, Nicolaus Copernicus University in Toruń, Jagiellońska 13-15, PL-85-067, Bydgoszcz, Poland; e Clinical Epidemiology Unit, Orthopedics, Department of Clinical Sciences Lund, Lund University, SE-221 85, Lund, Sweden

**Keywords:** Hip, Osteoarthritis, Arthroplasty, Replacement, HOOS, Outcome

## Abstract

**Objectives:**

Our objective was report short-term results of total hip replacement (THR) and to identify patients with functional recovery and with treatment failure. We also aimed to investigate whether there are any potential predicting factors for functional recovery or treatment failure.

**Design:**

Prospective cohort study. Clinical examination was performed and data were collected from patients before THR and at three follow-up assessments within subsequent year. The primary endpoint was the change between assessments in the average score on four subscales of the Hip disability and Osteoarthritis Outcome Score (HOOS_4_) covering pain, symptoms, activity of daily living, and quality of life. Secondary endpoints included results on all five HOOS subscales, and the SF–36. Functional Recovery and Treatment Failure were defined basing on the published reference population values.

**Results:**

We assessed 179 patients (98 women and 81 men, mean age at THR 67 years, range 31–90 years). The mean HOOS_4_ scores continued to improve up to 12 months after THR. Functional Recovery was identified in 32% while Treatment Failure in 16% of the patients. We found an association between high (>30.3) preoperative SF–36 Physical Component Summary scores and Functional Recovery, as well as low preoperative SF–36 Physical (<30.3) and Mental (<35.9) Component Summary scores and Treatment Failure.

**Conclusions:**

Knowing that only one third of subjects undergoing THR achieved Functional Recovery and one sixths had Treatment Failure, gives us a better perspective to discuss feasibility of expectations and, consequently, to prevent patient dissatisfaction following THR.

## Teaser

Trial registration number: Clinicaltrials.gov: NCT04691466.

## 1 Introduction

Total hip replacement (THR) is one of the most common and most successful surgical operations in medicine [1]. THR is undertaken to relieve pain and restore function to the hip joint with end-stage osteoarthritis (OA) [2]. Traditionally, the outcome of THR has been quantified by orthopaedic surgeons as morbidity and mortality rates, alternatively as implant survival rate indices [ [3,4]]. The 10-year survival of the THR implant was reported to be as high as 95% and 15-year survival over 85% [5]. The implant survival index, however, says nothing about postoperative patient satisfaction, pain reduction, hip function or quality of life. It has been estimated that between 6 and 15% of patients undergoing THR have persistent pain and functional limitation [6] and 6–7% are not satisfied with the result of the operation after one year [ [7,8]]. Since patients are generally less satisfied with their outcomes than surgeons [9], assessments made from the patient's perspective with patient-reported outcome measures (PROMs) are advocated [10]. PROMs are now commonly used in arthroplasty registers [11]. Most of the studies involving THR patients THR, even those based on registers, report patients' outcomes in a longer time perspective [12]. It has been found, however, that most of clinically relevant improvement following primary joint replacement occurs within the first six months postoperatively [ [13,14]] and that further progress on perceived physical functioning (but not on daily activities) can possibly be expected even beyond this period [ [[14], [15], [16], [17]]]. One-year follow-up scores are supposed to be two to three times higher than the baseline scores assessed before THR in different PROMs [ [18,19]]. Although the population reference values for both Oxford and Harris Hip Scores (OHS, HSS) [20] and Hip disability and Osteoarthritis Outcome Score (HOOS) [21] have recently been published, they have not yet been referred to patients' results after THR.

There is no consistency about what factors affect specific outcomes following THR [12].

It is known that worse preoperative function is associated with larger postoperative improvement [ [[22], [23], [24]]]. However, patients with low baseline scores do not reach the level of postoperative functioning that subjects with better preoperative function do [25]. The results concerning the role of potential predictors such as comorbidity, pain and preoperative health-related quality of life, educational level, patient expectations and mental well-being are conflicting [12].

To our knowledge, no study has characterized results of THR based on reference population data for the HOOS. Thus, our primary purpose was to report short-term THR results and identify patients with good outcome corresponding to functional recovery, as well as patients with unsatisfactory results that can be consistent with treatment failure. The secondary purpose was to investigate whether there are any potential predicting factors for functional recovery or treatment failure after THR.

## 2 Methods

### 2.1 Study design

This is a prospective cohort trial in patients participating in the Study Evaluating the Efficacy of Joint Replacement (SEVERE). The study is registered on ClinicalTrials.gov (NCT04691466).

### 2.2 Patients

Patients who had undergone THR at the Department of Orthopaedics, Ministry of the Interior and Administration Hospital in Olsztyn, Poland, between April 2013 and April 2016 were included in the study. All the patients had either primary or secondary hip OA, according to the American College of Rheumatology criteria [26], with indication for THR.

Exclusion criteria were: 1) inability to understand the Polish language, 2) presence of neuromuscular disease and cognitive impairment, and 3) having a prosthesis in another joint of the ipsilateral or contralateral lower limb placed within 6 months before THR surgery, 4) having rheumatoid arthritis, and 5) symptoms in several joints (hip, knee or ankle) with expected total joint arthroplasty within 1 year.

Surgical procedure and strategy of the postoperative management are presented in Additional File 2.

### 2.3 Outcome measures

Condition-specific scores were assessed using the Hip disability and Osteoarthritis Outcome Score (HOOS). The HOOS is a 40-item questionnaire which consists of five subscales: Pain, other Symptoms, Function in Daily Living (or Activity in Daily Living, ADL Function), Sports and Recreation Function and hip related Quality of Life (QOL)*.* A normalized score from 0 (indicating extreme problems) to 100 (indicating no problems at all) is calculated separately for each subscale.

The user's guide can be downloaded from www.koos.nu [27]. The Polish version of the HOOS is considered to be a valid and reliable measure in the assessment of patient-relevant outcome in subjects undergoing THR [28].

General health quality of life was assessed by the 36-item Short Form Health Survey (SF-36) that has already been validated in Polish [29]. Scores from 0 (worst possible health status) to 100 (best possible health status) were independently generated for Physical Component Summary and Mental Component Summary.

SF-36 outcomes were calculated with Scoring Software v. 4.5 delivered by the copyrights holder (Optum Insight, Eden Prairie, MN, USA, license number QM018125). The Physical and Mental Summary Scores were calculated according to the oblique rotation analysis. The oblique model was more reasonable than orthogonal rotation model [30] since it permits the assumption that mental and physical health interact with each other [31].

The intensity of hip pain was measured with the visual analog scale (VAS). The 100-mm VAS is a unidimensional scale and it is considered valid and reliable [32].

### 2.4 Data collection

Data were collected prospectively at four time points: before THR (at baseline, assessment A), and at routine follow-up 6–8 weeks after THR (assessment B), 6 months after THR (assessment C) and, finally, 12 months after THR (assessment D).

The preoperative (baseline) and follow-up assessments were done in the clinic. During assessment A, the participants were asked to fill out the Polish version of HOOS, the SF-36, the VAS for pain, and give data on sex, age, number of joints affected by arthritis, level of education, and occupational status (Additional File 3). At the follow-up assessments, the participants were asked to fill out the HOOS questionnaire.

All self-reported questionnaires, demographics and relevant information were processed by one orthopedic surgeon (MKG).

### 2.5 Primary outcome

#### 2.5.1 HOOS_4_ score change

The primary outcome was the change between the HOOS_4_ assessments, with the scores ranging from 0 (worst) to 100 (best). The HOOS_4_ is defined as an average score of the four HOOS subscale scores: HOOS Pain, HOOS Symptoms, HOOS ADL, and HOOS QOL. The fifth subscale, Sports and Recreation Function is excluded to avoid a floor effect, since hardly any elderly THR patients undertake sports activities [33].

### 2.6 Secondary outcomes

Secondary outcomes included results of all five HOOS subscales, the scores of the SF-36 Physical Component Summary, and Mental Component Summary and pain intensity in VAS.

For the purpose of the analysis, two concepts: ‘Functional Recovery’ and ‘Treatment Failure’ have been created.

### 2.7 Functional Recovery and Treatment Failure

We have specified two categories of the HOOS outcome for the study group: ‘Functional Recovery’ or ‘Treatment Failure’. Based on the published Swedish population reference, the Functional Recovery was defined as the score above the lower threshold for the 95% CI in every single HOOS subscale in respective age groups (18–34, 35–54, 55–74 and over 75 years), separately for men and women [21].

Treatment Failure was defined as a HOOS QOL score <44 which is a prespecified cutoff value consistent with a report of more than a moderately decreased hip-related quality of life.

### 2.8 Statistical analysis

Descriptive statistics were used to describe sociodemographic and clinical characteristics preoperatively, at baseline, and clinical characteristics after treatment, at follow-up. Continuous data were described using means with standard deviations (SD) and medians. The Shapiro-Wilk test was used to determine if the data were normally distributed. If the normality assumptions were met, parametric tests were used: independent or paired Student's t-test for quantitative variables and McNemar or Chi^2^ test for qualitative variables. When the normality assumptions were not satisfied, Mann-Whitney *U* test for continuous variable and the Fisher's exact or McNemar test for binary variables were used.

For the analysis of HOOS_4_, a linear mixed model (LMM) has been chosen. The reason is that with repeated values for the HOOS_4_ variable, there is a dependency between values for each individual. With a LMM approach, it is possible to include each individual with his or her own “path” while still having a linear regression model. The model has been set up with HOOS_4_ as the dependent variable, while timepoints, sex, age, Physical Component Summary, Mental Component Summary and BMI (the last four measured at baseline) as independent variables. For details about the covariance structure and other technical details, see the SPSS syntax (Additional File 4).

For the HOOS score, 1.54% of individual items were missing at Assessment A, 2.94% at B, 1.93% at C and 2.34% at D. A total score could be calculated for all subscales for 99% of the patients. Missing data were handled according to the HOOS scoring instructions [27] with the participate mean substitution method. Missing data were subsequently imputed with the mean of the other values within the same subscale. In order to quantify the difference in outcome change in respective HOOS subscales, a calculation of effect size was carried out. Effect size was computed as a mean score change divided by baseline SD (Kazis’ effect size) [34].

For the comparison of the HOOS_4_ scores at the four timepoints, marginal means have been computed. More specifically, this has been done with the implementation of such means in SPSS called “estimated marginal means”.

In order to determine whether the preoperative (baseline) evaluations were predictive of achieving either Functional Recovery or Treatment Failure, logistic regression models were created.

A number of independent continuous and categorical variables were examined. Patient-reported pain (from a visual analogue scale, VAS) was a continuous predictor. Subjects were divided according to sex and body mass index (BMI) and categorized as normal (<25), overweight (25–29.9) and obese (≥30). We also categorized individuals into tertiles based on their age (T1: <62.2 years, T2: 62.2–71.6 years, and T3: >71.6 years), scores in the Physical Component Summary (T1: <25.2, T2: 25.2–30.3 and T3: >30.3), and in the Mental Component Summary (T1: <35.9, T2: 35.9–44.3 and T3: >44.3) of the SF-36.

Missing data in all variables in regression analyses were imputed using the predicted value from an imputation model with the full conditional specification regression method for any missing pattern. Six imputed datasets were created, then the results were pooled in order to obtain a set of final parameter estimates that consequently were used in regression analyses [35].

Each independent variable was analyzed in an unadjusted and an adjusted model. The adjustment was made for age, sex, preoperative BMI, preoperative pain, Physical Component Summary and Mental Component Summary. The odds ratio (OR) estimates with 95% confidence intervals (95% CIs), and results from the likelihood ratio test, expressed as *p* values, were based on the models. Model fit was examined using the Hosmer-Lemeshow test [36].

All analyses were performed with the use of IBM SPSS Statistics for Windows v. 25.0.0 (IBM Corp. Armonk, New York, USA). We considered a two-tailed *p* value less than 0.05 to be significant. Sample size estimation was made with G∗Power software v. 3.1.9.7 (Universität Düsseldorf, Germany) [37] (Additional File 1).

## 3 Results

**Fig. 1:**
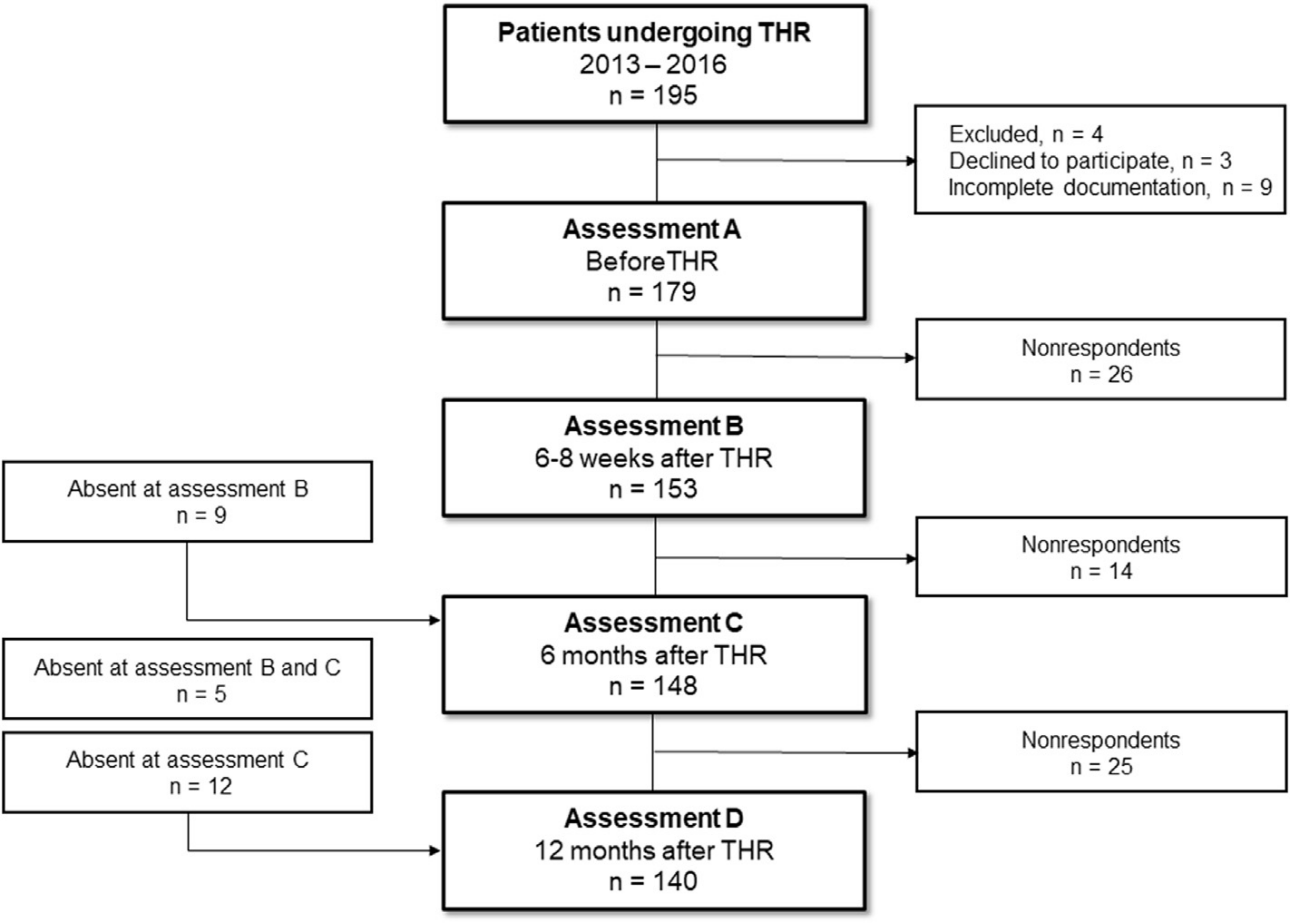
Flowchart presenting the study group formation.

Out of 195 subjects who underwent THR, 179 (92%) met the criteria and agreed to participate. At consecutive assessments, there were 153 (assessment B, 79%), 148 (C, 76%) and 140 patients (D, 72%) (Fig. 1).

The study group consisted of 98 women (55%) and 81 men (mean age 67 (31–90), median 66 years). Women were significantly older than men (mean [SD] 70 [9] years, vs. 64 [11] years, *p* ​< ​0.0001). One hundred and fifty-three patients (86%) were overweight or obese.

The patient characteristics are summarized in Table 1.

**Table 1:** Characteristics of the study group.

Characteristics	All subjects	Men	Women
	n ​= ​179	n ​= ​81	n ​= ​98
Age, mean (SD), years			
Assessment A	66.9 (10.6)	63.7 (11.3)	69.6 (9.3)
BMI, mean (SD)	29.5 (4.4)	30.0 (4.3)	29.1 (4.4)
BMI, n (%)			
Normal	26 (15)	8 (10)	18 (18)
Overweight	81 (45)	36 (44)	45 (46)
Obese	72 (40)	37 (46)	35 (36)
SF-36, mean (SD)			
Physical Component Summary	28.1 (6.4)	29.2 (5.9)	27.3 (6.7)
Mental Component Summary	39.5 (10.2)	40.7 (10.0)	38.5 (10.4)

Abbreviations: BMI – body mass index; SD – standard deviation. SF-36 – 36-item Short Form Health Survey.

BMI was categorized as normal (<25), overweight (25–29.9) and obese (≥30). Percentage values were rounded to integers (whole numbers).

### 3.1 Patients lost to follow-up

Out of 179 patients who attended assessment A, 39 (22%) were lost to the final follow-up (assessment D). To evaluate a possible inclusion bias, the subjects who participated at assessment D and those who did not, were analyzed with regard to age, sex, BMI (Table 1), occupation activity and educational level (Additional File 3). We found no significant differences between these characteristics (data not shown).

### 3.2 Primary outcome

#### 3.2.1 HOOS_4_

**Fig. 2:**
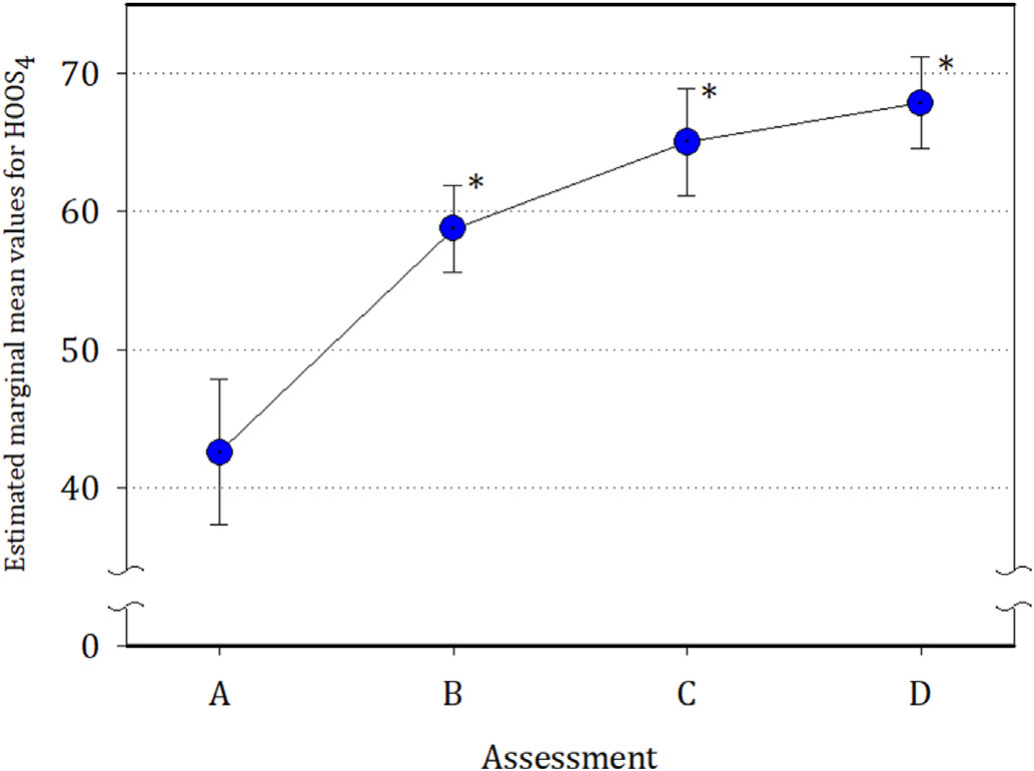
Estimated marginal means of the HOOS_4_ scores at the four time points (A to D). Circle midpoints represent mean HOOS_4_ scores. Assessments were made at entry (preoperatively, assessment A), then 6–8 weeks after THR (B), 6 months after THR (C) and 12 months after THR (D). Vertical bars represent 95% confidence interval (95%CI) values. Asterisks (∗) show *p* values ​< ​0.001.

The mean HOOS_4_ scores improved substantially within first six weeks after THR (at assessment B) and continued to improve in consecutive assessments (C and D) (*p* ​< ​0.0001 between following assessments). The score change between assessment A and D was 49.4 points producing a very large effect size of 2.47 (Fig. 2, Table 2).

**Table 2:** Scores in HOOS subscales and in VAS Pain in consecutive assessments: A (before THR), B (6–8 weeks after THR), C (6 months after THR) and D (12 months after THR).

HOOS Subscales		Assessment				*p*-values				ES (A vs. D)
		A	B	C	D	A vs. B	B vs. C	C vs. D	A vs. D	
**Pain**	Mean	31.2	70.7	77.2	80.3	<0.001	0.001	0.046	<0.001	3.19
	Median	30	70	82.5	85					
	SD	15.5	19.9	19.9	17.7					
	Range	0–82.5	22.5–100	27.5–100	22.5–100					

**Symptoms**	Mean	28.7	69	73.9	79.8	<0.001	0.012	0.003	<0.001	2.93
	Median	25	75	80	85					
	SD	17.9	21.1	21.9	18.2					
	Range	0–75	10–100	5–100	20–100					

**ADL**	Mean	26.5	63	73.3	76.8	<0.001	<0.001	<0.001	<0.001	3.10
	Median	25	63	79	81					
	SD	16.2	20.6	21.4	19.5					
	Range	0–68	15–100	18–100	19–100					

**Sport/Recreation**	Mean	14.2	42.3	58	59.8	<0.001	<0.001	0.105	<0.001	3.06
	Median	12.5	37.5	62.5	60.4					
	SD	14	27	30	28.3					
	Range	0–62.5	0–100	0–100	6–100					

**QOL**	Mean	18	50.6	61.3	64.7	<0.001	<0.001	0.028	<0.001	2.96
	Median	19	50	63	67					
	SD	15.5	21.2	23.3	22.3					
	Range	0–63	6–94	0–100	6–100					

**HOOS**_**4**_	Mean	26	63.2	71.5	75.4	<0.001	<0.001	<0.001	<0.001	2.47
	Median	26.2	63.2	76.1	79.6					
	SD	14.4	18.5	20.2	18					
	Range	0–67	18–97.5	18–100	19–100					

**VAS Pain**	Mean	6.8	0.9	0.8	0.4	<0.001	0.197	0.015	<0.001	3.25
	Median	7	0	0	0					
	SD	1.6	1.5	1.5	1.1					
	Range	1–10	0–7	0–7	0–5					

Abbreviations: HOOS – Hip disability and Osteoarthritis Outcome Score; THR – total hip replacement; ADL – activities of daily living; QOL – quality of life; ES – effect size.

*P*-values for the comparison between the results in consecutive assessments: A and B, B and C, C and D as well as A and D. Kazis' effect size (ES) was used for the comparison between results in assessment A and D.

### 3.3 Secondary outcomes

#### 3.3.1 Change in the HOOS subscales

The differences in mean scores of all the HOOS subscales between consecutive assessments were statistically significant except for the difference in the HOOS subscale Sports and Recreation Function between assessments C and D. Score changes between assessment A and D exceeded 45 points for all subscales (*p* ​< ​0.0001, effect sizes between 2.93 for HOOS Symptoms and 3.19 for the HOOS Pain) (Table 2).

### 3.4 Pain

The mean preoperative VAS score (assessment A) was 6.83 (range 1–10, median 7). The pain score was higher in women than in men (mean [SD] 7.15 [1.61] vs. 6.44 [1.61], *p* ​= ​0.002). Postoperatively, the VAS score was not remarkably different between assessments B and C but lower at D (mean 0.41 [1.07]). Postoperative differences between men and women were not significant (*p* ​= ​0.783 ​at assessment B, 0.838 ​at ​C, and 0.721 ​at D).

### 3.5 Functional Recovery

Functional Recovery was achieved in 41 out of 127 subjects (32%) who completed the HOOS questionnaire at assessment D. There was no significant difference in proportion of patients with Functional Recovery in men (14/57, 25%) and women (27/70, 39%) (*p* ​= ​0.127). All subjects with Functional Recovery improved between assessment A and D (data not shown).

Patients who had higher baseline scores (>30.3, tertile 3) in the Physical Component Summary were more likely to achieve Functional Recovery than those who scored <25.2 (tertile 1) in both models: unadjusted (OR ​= ​2.9; 95% CI 1.1–7.5) and adjusted model (OR ​= ​3.2; 95% CI 1.0–9.9). No association between age and Functional Recovery was observed (Table 3).

**Table 3:** Odds ratios for functional recovery after THR (at assessment D) by patient characteristics and selected predictors at assessment A.

Factor	Number of cases		Unadjusted model		Adjusted model	
	n	%	OR	95% CI	OR	95% CI
**Sex**						
Man^a^	57	45				
Woman	70	55	1.9	0.9–4.2	2.4	1.0–6.0
**Age**						
T1^a^	42	37				
T2	43	31	1.5	0.6–4.0	1.6	0.5–4.6
T3	42	32	2.2	0.9–5.6	2.2	0.8–6.6
**BMI**						
Normal	21	17	1.2	0.4–3.6	0.6	0.2–2.2
Overweight	55	43	1.3	0.6–2.9	1.0	0.4–2.5
Obese^a^	51	40				
**PCS**						
T1^a^	42	33				
T2	42	33	1.6	0.6–4.4	1.7	0.6–5.0
T3	43	34	2.9	1.1–7.5	3.2	1.0–9.9
**MCS**						
T1^a^	42	33				
T2	42	33	1.0	0.4–2.6	0.9	0.3–2.7
T3	43	34	2.2	0.9–5.6	2.0	0.7–6.1
**VAS**						
	125	100	0.9	0.7–1.1	1.0	0.7–1.2

Abbreviations: THR – total hip replacement; BMI – body mass index; PCS – physical component summary; MCS – mental component summary; OR – odds ratio; 95% CI – 95% confidence interval; T – tertile.

BMI was categorized as normal (<25), overweight (25–29.9) and obese (≥30). Subjects were categorized into tertiles according to the distribution of age (T1: <62.2 years, T2: 62.2–71.6 years, and T3: >71.6 years) to the baseline PCS scores (T1: <25.2, T2: 25.2–30.3 and T3: >30.3) and MCS scores (T1: <35.9, T2: 35.9–44.3 and T3: >44.3). OR and 95% CI values were rounded to decimals.

a Reference category.

### 3.6 Treatment Failure

Treatment Failure was observed in 20 out of 127 (16%) subjects at assessment D. No difference in the proportion of men (10/57, 18%) and women (10/70, 14%) was observed (*p* ​= ​0.81). All patients with Treatment Failure improved in HOOS_4_ between assessment A and D.

Patients with preoperative Physical Component Summary values ​> ​30.3 (tertile 3) were estimated to be less likely to have a Treatment Failure than those belonging to tertile 1 in both unadjusted (OR ​= ​1/0.067 ​= ​14.9) and adjusted model (OR ​= ​1/0.097 ​= ​10.3) (Table 4). Assuming that preoperative score >30.3 is the reference level category, patients with values ​< ​25.2 (tertile 1) were more likely to have Treatment Failure than those belonging to tertile 3 (OR ​= ​14.9, 95% CI 1.8–121.6 in the unadjusted model and OR ​= ​10.3, 95% CI 1.0–100.5 in the adjusted model). In addition, having preoperative Physical Component Summary score within tertile 2 (25.2<PCS<30.3) was a factor associated with Treatment Failure in the unadjusted model (OR ​= ​9.9; 95% CI 1.2–82.9).

**Table 4:** Odds ratios for Treatment Failure after THR (at assessment D) by patient characteristics and selected predictors at assessment A.

Factor	Number of cases		Unadjusted model		Adjusted model	
	n	%	OR	95% CI	OR	95% CI
**Sex**						
Man^a^	57	45				
Woman	70	55	0.8	0.3–2.0	0.3	0.1–1.0
**Age**						
T1^a^	42	37				
T2	43	31	0.3	0.1–1.3	0.3	0.1–1.5
T3	42	32	1.2	0.4–3.4	1.4	0.4–5.2
**BMI**						
Normal	21	17	1.7	0.5–5.9	3.0	0.6–14.5
Overweight	55	43	0.8	0.3–2.3	0.8	0.2–2.7
Obese^a^	51	40				
PCS						
T1^a^	42	33				
T2	42	33	0.7	0.2–1.9	0.7	0.2–2.2
T3	43	34	0.1	0–0.6	0.1	0–1.0
MCS						
T1^a^	42	33				
T2	42	33	0.3	0.1–0.9	0.2	0.1–0.9
T3	43	34	0.3	0.1–0.9	0.3	0.1–1.2
VAS						
	125	100	1.2	0.9–1.2	1.1	0.8–1.7

Abbreviations: THR – total hip replacement; BMI – body mass index; PCS – physical component summary; MCS – mental component summary; OR – odds ratio; 95% CI – 95% confidence interval; T – tertile.

BMI was categorized as normal (<25), overweight (25–29.9) and obese (≥30). Subjects were categorized into tertiles according to the distribution of age (T1: <62.2 years, T2: 62.2–71.6 years, and T3: >71.6 years) to the baseline PCS scores (T1: <25.2, T2: 25.2–30.3 and T3: >30.3) and MCS scores (T1: <35.9, T2: 35.9–44.3 and T3: >44.3). OR and 95% CI values were rounded to decimals.

a Reference category.

Patients with preoperative Mental Component Summary values ​> ​44.3 (tertile 3) were less likely to have a Treatment Failure than those with values ​< ​35.9 (OR ​= ​1/0.256 ​= ​3.9) in an unadjusted model. Patients from tertile 2 (preoperative values between 35.9 and 44.3) were less likely to have a Treatment Failure than those belonging to tertile 1 in both unadjusted (OR ​= ​1/0.263 ​= ​3.8) and adjusted model (OR ​= ​1/0.214 ​= ​4.7) (Table 4). The other way around, preoperative Mental Component Summary <35.9 was a factor significantly associated with confirming Treatment Failure in unadjusted (OR ​= ​3.9; 95% CI 1.1–13.3) analysis.

## 4 Discussion

Our study aimed to prospectively document the improvement over 12 months in patients who had undergone THR as well as to assess Functional Recovery and Treatment Failure in these patients.

We found that patients after THR improved in both HOOS_4_ and in separate HOOS subscales. The mean difference between the baseline results (at assessment A) and the HOOS scores one year after THR (at assessment D) is nearly 50 points in the HOOS_4_, and even more than 50 points in two HOOS subscales: Symptoms and ADL. This remarkable improvement was higher than reported in other studies [ [38,39]]. However, the preoperative scores in our study were between 14 points in the HOOS subscale Sports and Recreation Function and 31 points in the subscale Pain, which was substantially lower than demonstrated in other studies.

Baseline HOOS results over 30 points in patients at similar age were reported by Nilsdotter et al. [33] and Arbab et al. [40]. In another study performed in slightly younger subjects who underwent THR with both short- and conventional stem implants, the baseline results of mean total HOOS score were 36 and 37 points, respectively, and over 25 points in the lowest-scored subscale Sport and Recreation Function [39].

The lower preoperative scores observed in our study could be explained by possible deterioration in physical function caused by longer waiting time for THR [41]. The time from referral to THR was 3 years in our study compared to less than 6 months in Denmark and 3 months in the Netherlands [42]. In addition, the mean BMI of our patients was higher than that reported by others [ [39,40]].

As we observed, most of the improvement occurred in the first 6 months, with the steepest recovery trajectory within 6–8 weeks after THR. This observation corresponds to earlier findings, i.e. that most of the improvement occurs within the first 3 months [ [43,44]]. According to the results of other studies, no [44] or only small clinical improvements [ [45,46]] can be expected beyond the first 3-month period.

To the best of our knowledge, there have been no studies to compare outcome in patients undergoing THR with hip-specific population-based reference data. We have based the two concepts of Functional Recovery and Treatment Failure on the Swedish population study of Sundén et al. [21]. The Functional Recovery level, defined as the lower threshold for the 95% CI of sex- and age-matched subjects from population-based reference data, was described earlier for patients undergoing anterior cruciate ligament reconstruction and has been assessed with the Knee injury and Osteoarthritis Outcome Score (KOOS) [47].

Our study showed that despite an incremental score gain in the HOOS_4_ and all HOOS subscales, only 32% of patients who attended the final follow-up at assessment D achieved a Functional Recovery status, consequently attained the hip condition equal to or even better than average in age- and sex-matching population.

Outcomes such as pain and function are typically strongly associated with the level of patient satisfaction. Since satisfaction reflects also factors not directly related to the health status [48], it must be regarded as only an indirect or a proxy indicator of the intervention quality [49]. Since patient satisfaction has been reported to be highly dependent on preoperative patient expectations [50] and baseline scores [51], it is likely that a low preoperative score is associated with greater room for improvement and thus greater patient satisfaction [51]. Consequently, it is not surprising that patients from our cohort, who generally scored relatively low before THR, reported very high levels of satisfaction [28].

In the present study we use a definition of Treatment Failure based on the one-year follow-up score in the HOOS subscale QOL. Traditionally, failure of THR is defined as an implant-related problem demanding revision and requires a long-term observation. The modes of failure include implant wear, aseptic loosening, dislocation, infection, and periprosthetic fracture to name the most frequent ones [52]. Using our approach, we observed Treatment Failure in 16% of participants. This relatively high rate of unsatisfactory results can be associated with a potential mode of implant failure that appears later on, beyond one-year follow-up.

We did not observe any association of age and either Functional Recovery or Treatment Failure. This observation is not in accordance with previous publications [ [[53], [54], [55]]] that reported smaller improvements or worse outcomes in older patients. This phenomenon can be neutralized by the fact that population-based reference values are lower for the oldest patients, which, in turn, makes these patients possible to score higher.

Data concerning the association between preoperative pain scores and outcome of THR are conflicting [ [12,44]]. Likewise, little is known whether preoperative functional scores can be useful in predicting recovery. It has been reported, however, that worse preoperative function is associated with larger postoperative improvement [ [12,51]], although patients with low function scores before THR do not achieve postoperative levels of those who scored higher at baseline [25]. Our work suggests that higher preoperative Physical Component Summary scores are associated with Functional Recovery while low Physical Component Summary scores predisposed to Treatment Failure at one-year follow-up, which is in line with the findings of Weber et al. [56]. However, due to the low specificity of the SF-36 towards hip complaints and the rather subtle differences between results in selected groups (tertiles), this observation should be interpreted with caution.

Even bigger interpretation concerns appear when analyzing the association of preoperative mental status and THR outcome. We found that low preoperative Mental Component Summary scores were predictive of Treatment Failure. Although it has previously been suggested that depression and worse mental well-being were related to worse outcome of THR [57,58], the predictive properties of Mental Component Summary were not confirmed [56]. Further research with use of more advanced assessment of depression and anxiety is needed to provide more reliable data that could help in proper patient selection and/or pre-THR treatment.

The application of proven and validated measurement tools, multiple follow-up assessments and a reasonable sample size with good power are the strengths of this study. In addition, the use of linear mixed regression models allowed the modelling of repeated measures in order to follow outcome changes and to characterize patient response over time.

Our study has some limitations. The research was designed as a single center study and was performed in patients from sparsely populated north-eastern Poland, thus our results may not be generalized to a larger population of the country. With its 22% loss to the final follow up at assessment D, the study can be regarded to have a high risk of bias; one can expect that loss to follow up is related to the missing not at random mechanism since patients who are doing well are less likely to return to follow-up assessments.

A slight female predominance with the female/male ratio of 1.2 and the higher average age of women undergoing THR reflects a well-known observation that women with OA undergo surgery at a later stage of the disease [8]. Despite discrete ethnic and cultural differences, we believe that the reference data from the Swedish population can even be applied in Poland. Since the sample size was relatively small, missing data, even if there were only few, reduced the power of our analyses. Missing values at the item level have been imputed successfully by mean substitution, a method which has its drawbacks, mainly that correlations between imputed variables are attenuated. However, this method was chosen because it was regarded as important to use the same protocol for the calculation of the HOOS scales as suggested by the original authors. As we assumed that the incomplete data at the variable level (for those covariates that were included in regression analyses) were missing at random, we have replaced them by multiple imputation using the full conditional specification method. This strategy allowed us to correct bias and substantially improve precision of calculations.

Based on data concerning objective postoperative evaluations and patient improvement observed in PROMs, clinicians can have an overrated picture of patients’ health after THR. Since the results of THR are associated with fulfillment of patient preoperative expectations (irrespective of whether they are high or low), setting the reasonable goals of treatment seems to be essential to achieve optimal postoperative results [59]. Knowing that only one third of subjects undergoing THR will reach population-based Functional Recovery, clinicians will have a better perspective to discuss rationality of expectations and, consequently, prevent patient dissatisfaction.

## Authors’ contributions

PTP conceived and designed the study. MKG was responsible for acquisition of data and creation of datasets. PTP and MKG drafted the manuscript. RL takes responsibility for the integrity of the data and the accuracy of the data analysis. The article was revised critically for important intellectual content by PTP. All authors participated in the analysis and interpretation of the data, and approved the final version for submission.

## Role of the funding source

This work was supported by the 10.13039/100012409Faculty of Health Sciences, Ludwik Rydygier Collegium Medicum of the Nicolaus Copernicus University, Toruń, Poland (grant WN 758), and by the 10.13039/501100009772County Council of Norrbotten, Sweden.

## Ethics approval and consent to participate

The study was conducted in accordance with the ethical standards of the institutional and/or national research committee and with the 1964 Helsinki declaration and its later amendments or comparable ethical standards. The patients were informed in writing and orally by the study personnel, and a written informed consent was obtained from all subjects. Participation was voluntary, and withdrawal was possible at any time. All patients signed and personally dated the informed consent forms at admission to hospital, before participating in the study. The study was approved by the Medical Ethics Committee of the Local Chamber of Physicians and Dentists of the Warmia and Mazury Province in Olsztyn (Approval no. 140/2013).

## Declaration of competing interests

The authors declare no conflict of interests.

## APPENDIX A ADDITIONAL FILES

1. Sample size estimation and power analysis
2. Surgical procedure and postoperative management
3. Occupation activity and educational level
4. SPSS syntax for the HOOS modelling
