# Supplementary material for: Short-term patient-reported outcomes following total hip replacement: Is the success picture overrated?
Source: Osteoarthr Cartil Open. 2021 Jun 15;3(3):100192. doi: 10.1016/j.ocarto.2021.100192 (PMC9718121; doi:10.1016/j.ocarto.2021.100192)
Supplement: Multimedia component 1 [file mmc1.docx]

## **SPSS syntax for the HOOS modelling**

### **Variables**

| age_A | Age at assessment A |
| --- | --- |
| VAS_A | Pain score at assessment A |
| VAS_B | Pain score at assessment B |
| VAS_C | Pain score at assessment C |
| VAS_D | Pain score at assessment D |
| PCS_OBL | Physical Component Summary score (oblique model) at assessment A |
| MCS_OBL | Mental Component Summary score (oblique model) at assessment A |
| HOOS4_A | HOOS4 score at assessment A |
| HOOS4_B | HOOS4 score at assessment B |
| HOOS4_C | HOOS4 score at assessment C |
| HOOS4_D | HOOS4 score at assessment D |
| gender | Gender of subject studied |

### **Syntax**

DATASET ACTIVATE data_wide.
DATASET COPY data_long.
DATASET ACTIVATE data_long.

VARSTOCASES
/MAKE hoos4 FROM HOOS4_A HOOS4_B HOOS4_C HOOS4_D
/MAKE vas FROM VAS_A VAS_B VAS_C VAS_D
/INDEX=index(4)
/KEEP=ID age_A gender BMI_A PCS_OBL MCS_OBL
/NULL=KEEP.

RECODE index (1=0) (2=6) (3=26) (4=52) INTO time.
EXECUTE.

VARIABLE LEVEL BMI_A vas time(SCALE).

VALUE LABELS index 1 'A' 2 'B' 3 'C' 4 'D'.

*Generalized Linear Mixed Models.
GENLINMIXED
 /DATA_STRUCTURE SUBJECTS=ID REPEATED_MEASURES=index COVARIANCE_TYPE=DIAGONAL
 /FIELDS TARGET=hoos4 TRIALS=NONE OFFSET=NONE
 /TARGET_OPTIONS DISTRIBUTION=NORMAL LINK=IDENTITY
 /FIXED EFFECTS=gender index age_A BMI_A PCS_OBL MCS_OBL vas USE_INTERCEPT=TRUE
 /RANDOM USE_INTERCEPT=TRUE SUBJECTS=ID COVARIANCE_TYPE=IDENTITY SOLUTION=FALSE
 /BUILD_OPTIONS TARGET_CATEGORY_ORDER=ASCENDING INPUTS_CATEGORY_ORDER=ASCENDING MAX_ITERATIONS=100 CONFIDENCE_LEVEL=95
DF_METHOD=RESIDUAL COVB=ROBUST PCONVERGE=0.000001(ABSOLUTE) SCORING=0 SINGULAR=0.000000000001
 /EMMEANS TABLES=index COMPARE=index CONTRAST=SIMPLE
 /EMMEANS_OPTIONS SCALE=ORIGINAL PADJUST=LSD.
