# Supplementary material for: Short-term patient-reported outcomes following total hip replacement: Is the success picture overrated?
Source: Osteoarthr Cartil Open. 2021 Jun 15;3(3):100192. doi: 10.1016/j.ocarto.2021.100192 (PMC9718121; doi:10.1016/j.ocarto.2021.100192)
Supplement: Multimedia component 2 [file mmc2.docx]

**Sample size and power analysis**

Since the HOOS_4_ has not yet been used in clinical studies and the minimal detectable change (MDC) is not established for this construct, the sample size estimation was based on the outcome in the HOOS Pain subscale [1]. We assumed that MDC is a 10 points change between the baseline (A) and the 12-month follow-up (D) assessments. Nilsdotter et al. found that the mean HOOS Pain score 6 months after THR was 82.3 with standard deviation (SD) 18.3 [2]. When these values were presupposed with a small expected effect size of 0.55, α error of 0.05 and statistical power of 99%, the calculations indicated that the sample size should be at least 57 subjects. To compensate for a possible 30% loss to follow-up at assessment D, 74 subjects should be included at baseline assessment. However, the number of participants should be bigger due to possible large discrepancies and SD values at entry.

A *post hoc* analysis showed that in the study group that consisted of 140 subjects who scored 80.3 (SD 17.7) in the HOOS subscale Pain at assessment D, effect size was 0.58. With MDC of 10 points and α error of 0.05, the statistical power was 0.999.

Sample size estimation was made with G*Power software v. 3.1.9.7 (Universität Düsseldorf, Germany) [3].

**REFERENCES**

[1] S. Lyman, Y. Y. Lee, A. S. McLawhorn, W. Islam, C. H. MacLean, What Are the Minimal and Substantial Improvements in the HOOS and KOOS and JR Versions After Total Joint Replacement?, Clin Orthop Relat Res, 476 (2018) 2432-2441. https://doi.org/10.1097/CORR.0000000000000456.

[2] A. K. Nilsdotter, L. S. Lohmander, M. Klassbo, E. M. Roos, Hip disability and osteoarthritis outcome score (HOOS)--validity and responsiveness in total hip replacement, BMC Musculoskelet Disord, 4 (2003) 10. https://doi.org/10.1186/1471-2474-4-10.

[3] F. Faul, E. Erdfelder, A. Buchner, A. G. Lang, Statistical power analyses using G*Power 3.1: tests for correlation and regression analyses, Behav Res Methods, 41 (2009) 1149-1160. https://doi.org/10.3758/BRM.41.4.1149.
