# Supplementary material for: Short-term patient-reported outcomes following total hip replacement: Is the success picture overrated?
Source: Osteoarthr Cartil Open. 2021 Jun 15;3(3):100192. doi: 10.1016/j.ocarto.2021.100192 (PMC9718121; doi:10.1016/j.ocarto.2021.100192)
Supplement: Multimedia component 3 [file mmc3.docx]

**Surgical procedure**

THR was performed with patients in the lateral decubitus position through the posterolateral approach. All patients were operated by two experienced orthopedic surgeons, including the first author (MKG). Patients received either cementless or cemented implants. The type of implant was chosen according to preoperative assessment of bone stock. In the cementless group, two different implants were used: Taperloc stem/Rim Cup (Biomet Inc., Warsaw, Indiana, USA) or EcoFit (Implantcast GmbH, Buxtehude, Germany). The implants that were applied in the cemented group were: Taperloc stem/ M.E. Müller Low Profile Cup (Biomet Inc., Warsaw, Indiana, USA) or EcoFit stem/Müller II PE Cup (Implantcast GmbH, Buxtehude, Germany). No wound drainage was used.

**Postoperative management**

All patients were given postoperative either low-molecular-weight heparin (enoxaparin sodium, dalteparin sodium) or novel oral [anticoagulants](https://www.dicardiology.com/channel/antiplatelet-and-anticoagulation-therapies) (dabigatran or rivaroxaban) and prophylactic antibiotic therapy (intravenous cefazolin or, in the case of penicillin/cephalosporin allergy, clindamycin).

All patients followed a standardized post-operative programme of physiotherapy that was started on the day of surgery and continued until discharge. Immediate mobilization was attempted with full weight bearing allowed.
